# Effects of Classroom-Based Resistance Training With and Without Cognitive Training on Adolescents’ Cognitive Function, On-task Behavior, and Muscular Fitness

**DOI:** 10.3389/fpsyg.2022.811534

**Published:** 2022-03-21

**Authors:** Katie J. Robinson, David R. Lubans, Myrto F. Mavilidi, Charles H. Hillman, Valentin Benzing, Sarah R. Valkenborghs, Daniel Barker, Nicholas Riley

**Affiliations:** ^1^Priority Research Centre for Physical Activity and Nutrition, College of Human and Social Futures, University of Newcastle, Callaghan, NSW, Australia; ^2^Hunter Medical Research Institute (HMRI), Newcastle, NSW, Australia; ^3^School of Education/Early Start, University of Wollongong, Wollongong, NSW, Australia; ^4^Department of Psychology, Northeastern University, Boston, MA, United States; ^5^Department of Physical Therapy, Movement, and Rehabilitation Sciences, Northeastern University, Boston, MA, United States; ^6^Institute of Sport Science, University of Bern, Bern, Switzerland

**Keywords:** executive function, resistance training, cognition, on-task behavior, cognitive demand

## Abstract

**Aim:** Participation in classroom physical activity breaks may improve children’s cognition, but few studies have involved adolescents. The primary aim of this study was to examine the effects of classroom-based resistance training with and without cognitive training on adolescents’ cognitive function.

**Methods:** Participants were 97 secondary school students (45.4% females, mean age 15.78 ± 0.44). Four-year 10 classes from one school were included in this four-arm cluster randomized controlled trial. Classes were randomly assigned to the following groups: sedentary control with no cognitive training, sedentary with cognitive training, resistance training without cognitive training, and resistance training with cognitive training. Sessions varied in levels of both cognitive demand and resistance training (i.e., high vs. low) and were administered three times per week for 4 weeks (12 sessions). Inhibition, cognitive flexibility, episodic memory, on-task behavior, and muscular fitness were assessed at baseline and post-test. Linear mixed models were used to examine changes within and between groups.

**Results:** In comparison with the control group, episodic memory improved significantly in the resistance training without cognitive training group (−9.87 units, 95% CI: −17.71 to −2.03, *p* = 0.014, *d* = 0.72). There were no group-by-time effects for inhibition or cognitive flexibility. Classroom activity breaks both with and without cognitive demand improved participants’ on-task behavior in comparison with the control and sedentary group. The resistance training programs did not lead to improvements in muscular fitness.

**Conclusion:** Participation in body weight resistance training without cognitive training led to selective improvements in episodic memory. No training effects were found for inhibition or cognitive flexibility. A longer study period may be necessary to induce improvements in muscular fitness and associated changes in inhibition and cognitive flexibility.

**Clinical Trial Registration:** https://www.anzctr.org.au/ACTRN12621001341819.aspx, Australian New Zealand Clinical Trials Registry—ACTRN12621001341819.

## Introduction

A growing body of evidence suggests a positive association between children’s physical activity and their cognitive function. Although the majority of research in this field has been conducted with preschool and primary school-aged populations (Egger et al., 2019; Mavilidi et al., 2020; Schmidt et al., 2020; Bedard et al., 2021), evidence suggests that participation in physical activity can improve cognitive function across school children of different ages. Cognitive functioning exists on a continuum from basic information processing at one end to high levels of executive function at the other (Blair and Raver, 2015). The three core executive functions include inhibition (i.e., the aspect of self-control that involves maintaining focus on relevant aspects of the environment and resisting temptations and not acting impulsively or prematurely), cognitive flexibility (i.e., adjusting to new demands, rules, or priorities), and working memory (i.e., holding information in mind and mentally manipulating it; Diamond, 2013). Similar to working memory, episodic memory is related to pre-frontal and hippocampal brain regions. The development of the episodic memory reflects a change in the capacity to form, store, and retrieve representations binding events to context and is therefore dependent on the regulation of memory processes (Ghetti and Lee, 2011).

Various types of interventions have been shown to foster the development of executive functions, including computer-based trainings, educational programs, and classroom-based physical activity breaks (Diamond and Lee, 2011; Watson et al., 2017; Tomporowski and Pesce, 2019). Of the existing strategies designed to promote executive functions, classroom-based physical activity breaks are perhaps the most attractive for schools because they can provide an important dose of physical activity and enhance student learning (Watson et al., 2017). Compared to sedentary classrooms, participation in short-active classroom breaks (e.g., 5–20 min)can improve students’ executive functioning (Graham et al., 2021). In addition, chronic studies examining the longer-term effects of classroom activity breaks have observed improvements in working memory (Schmidt et al., 2020), inhibition (Pesce et al., 2016), and cognitive flexibility (Pesce et al., 2013). Previous studies have largely focused on the quantitative (e.g., exercise duration and intensity) characteristics of physical activity breaks, while the emphasis has now shifted to qualitative (e.g., type of exercise and task complexity) aspects (Pesce, 2012).

Increasing the cognitive demand of a physical activity program is one qualitative strategy to specifically target the development of core executive functions. Increased cognitive demand is thought to intensify cognitive engagement, requiring participants to exert greater cognitive effort in order to perform difficult tasks (Tomporowski et al., 2015). This could be achieved using sequential training, where exercise and cognitive training are undertaken separately or simultaneous or dual-task training, where cognitive training is performed simultaneously with exercise (Tait et al., 2017). It is difficult to draw firm conclusions about the benefits of cognitively demanding physical activity due to the limited number of studies and inconsistent findings (Singh et al., 2019; Leahy et al., 2020). Some studies report that increased cognitive demand has no impact on executive function (Mavilidi et al., 2020; Bedard et al., 2021). Other studies comparing cognitively challenging to non-cognitively challenging physical activity have shown that interventions, such as tag games, team games, or coordinative exercises, have a positive effect on executive functions (Chang et al., 2013; Schmidt et al., 2015; van der Niet et al., 2016). However, these latter studies failed to disentangle the physical and cognitive demands of the interventions. One study in children aged 7–9 years used keywords during games, requiring students to respond by matching the key word to the associated correct movement (Egger et al., 2019). The level of difficulty in the game increased incrementally and required participants to update new information (working memory), inhibit previous responses (inhibition), and shift between the keywords and their associated exercises (cognitive flexibility). Another study delivered 12 games to children aged 4–6, each of which specifically detailed the cognitive requirement (Schmidt et al., 2020). Although increasing the cognitive challenge embedded within a physical activity is one approach to increasing cognitive demand, the practicality of implementing this in a school setting on a regular basis presents several challenges.

Type of physical activity is another qualitative characteristic that requires further exploration. Classroom exercise breaks typically aim for a target intensity rating of moderate-to-vigorous and most studies attempt to achieve these objectives through aerobic exercises (Daly-Smith et al., 2018). Alternatively, resistance training has successfully been used in combination with aerobic exercise to gain improvements in executive functions (So and Kim, 2015), but few studies have examined the effects of resistance training in isolation. Body weight exercise is a scalable form of resistance training that does not require equipment. Evidence suggests that body weight exercise can be used successfully to improve musculoskeletal fitness and general health in adults (Voss et al., 2011), although less is known about the utility of this type of training in adolescent populations (Faigenbaum and Myer, 2010; Kennedy et al., 2018). A meta-analyses of 16 longitudinal datasets indicated resistance training had a positive effect on measures of executive function in adults (*d* = 0.39), while in a separate analysis of the effect of resistance training on working memory was not significant (*d* = 0.15; Landrigan et al., 2019). In a study that compared the effects of three types of physical activity (aerobic, coordination, and strength exercises) on the cognitive performance of children no effect on cognition was found (Van den Berg et al., 2016). All three groups recorded low exercise intensity ratings, which may explain the absence of differential effects between groups and possibly the effects on cognition which contradict previous findings, particularly for aerobic exercise (Van den Berg et al., 2016).

Our study was designed to investigate the impact of body weight resistance training with and without additional cognitive training on participants’ cognitive functions, on-task behavior, and muscular fitness. We hypothesized that participants assigned to the *resistance and cognitive training* group would experience larger improvements in cognitive functions than participants in the *resistance training*, *cognitive training*, and *control* groups. We also predicted that participants in the *resistance training* and *cognitive training* groups would experience larger improvements in cognitive function than participants in the control group. We hypothesized that participants in the *resistance and cognitive training* group and the *resistance training* only group would spend more time on task compared to participants in the *cognitive training* and *control* groups. Finally, we hypothesized that participants in both *resistance training* groups would experience improvements in muscular fitness.

## Materials and Methods

### Design

Approval was sought and obtained from the University of Newcastle Human Research Ethics Committee (H-2019-0415), and the Catholic Schools Office in the Maitland-Newcastle Diocese to conduct this study. We conducted a four-arm cluster randomized control trial design to compare the following experimental conditions: sedentary control with no cognitive training (CON), sedentary with cognitive training (SECT), resistance training without cognitive training (RTNC), and resistance training with cognitive training (RTCT). This approach is a non-equivalent groups design with students randomized at the class level due to school timetable constraints. Data were collected at baseline and post-test (4 weeks) for two aspects of executive function (inhibition and cognitive flexibility), episodic memory, on-task behavior, and muscular fitness. An acute rating of perceived exertion of the four conditions (i.e., CON, SECT, RTNC, and RTCT) was recorded before and after each activity break. This study was registered with the Australian New Zealand clinical Trials Registry: ACTRN12621001341819.

### Participants

**Figure 1:**
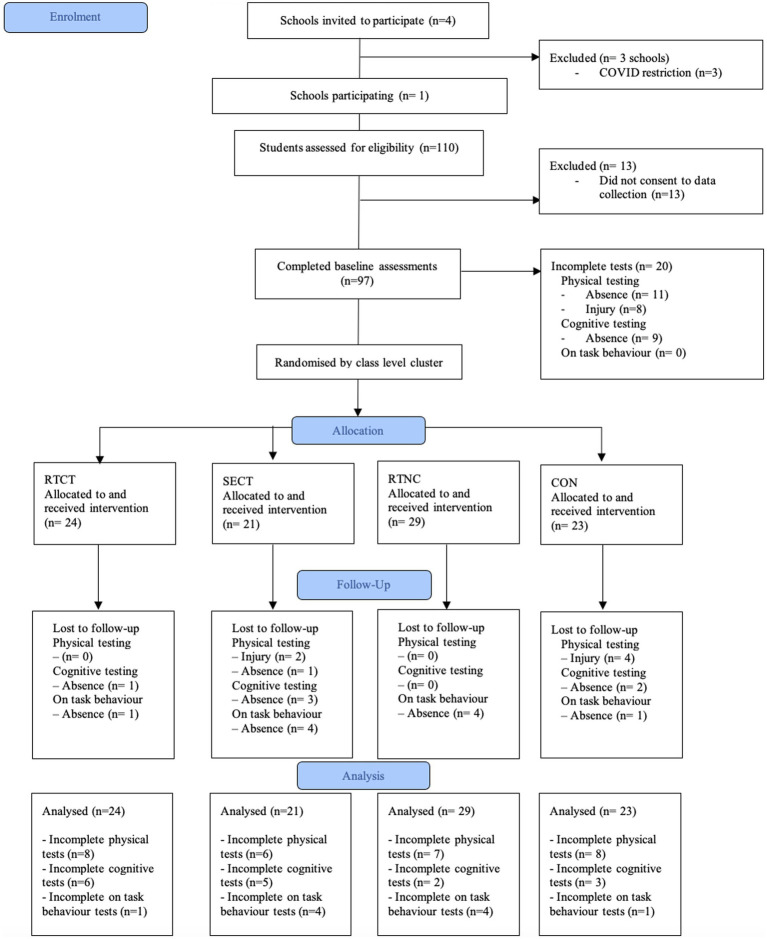
Consort flow diagram of participants.

Four high schools located in Maitland, NSW, Australia, were recruited to participate in this study. However, the impact of COVID-19 restrictions and changes to “outside researcher” policy enforced by the schools governing body (The Catholic School Office) reduced the permitted number to one. The school principal and classroom teachers of one school agreed to participate under strict COVID protocols, resulting in 4-year 10 mathematics classes being assigned to the study. To align with school procedures, the intervention would be delivered to the whole class. All students were eligible to participate (*n* = 110), but only students who returned the consent form completed the assessments (*n* = 97, 53 males). Following baseline testing, classes were randomly assigned to one of the four experimental conditions: CON (*n* = 23), SECT (*n* = 21), RTNC (*n* = 29), and RTCT (*n* = 24). See Figure 1 for the flow diagram of participants.

### Procedure

The classroom activity breaks were administered as pre-recorded videos at the start of mathematics lessons. Mathematics was selected to ensure consistency across participants and classes. Each video was designed as a lesson starter, being delivered three times per week, for 4 weeks (12 sessions in total). Prior to the beginning of the study, a 3-h training session was delivered to the participating teachers by the lead author outlining their role in the intervention. The protocol components focused on teacher delivery (i.e., video projection at the beginning of the lesson), motivation techniques for encouraging student participation and management of potential injured students. Classrooms were inspected before the study began to confirm that students could safely complete all tasks. Multimedia displays were checked to ensure that all students could clearly see the screen from the back of the classroom. No alterations were needed for any of the classrooms.

**Figure 2:**
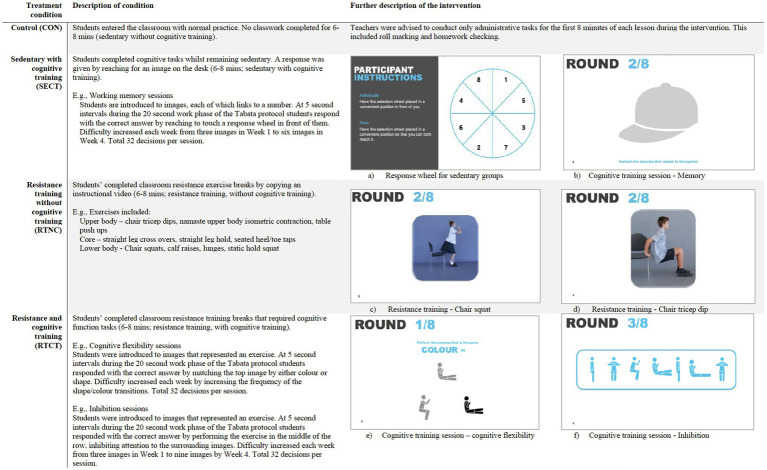
Outline of intervention group activities.

The qualitative focus of the videos was to deliver dual-task challenges that targeted each executive function separately. Each video was presented to the whole class and used a 2-min introduction where students were introduced to the cognitive challenge focus of the day (e.g., inhibition or cognitive flexibility). Each week the level of cognitive demand experienced by the cognitive training groups increased, reflecting a repetitious and challenging intervention. See Figure 2 for a detailed summary and example of the intervention components per condition.

The video progressed with exercises to target muscular strength and used a Tabata format of 20 s work/10 s rest × 8 sets, with a total workout time of 4 min. To increase the cognitive training and maintain engagement, the 20 s work phase was split into 4 × 5 s exercises. Students in the cognitive training groups were required to make 32 decisions per session.

Each condition performed the following activities and cognitive tasks:

– In the CON group, students enter the classroom with normal practice. No classwork was completed, rather students completed administration activities, e.g., roll marking and homework checking for 6–8 min.
– The SECT group would respond to the video stimulus in their sedentary position by selecting an image on the response wheel on their desk in front of them.
– In the RTNC group, the cognitive demand was removed as students simply copied the set of resistance training exercises shown on the intervention video.
– The RTCT group would respond to the question on the video stimulus by performing the associated resistance training exercise (e.g., one session totals 32 × 5 s of resistance training activities).

All exercises were performed at the desk and included table push-ups, chair triceps dips, and alternate lunges and squats. Isometric holds were also incorporated into the muscular fitness work out offering variety to students and a unique approach to resistance training in limited space. The two resistance training groups (RTNC and the RTCT) performed the same body weight exercises. The cognitive activities were also the same in the cognitive training groups (RTCT and RTNC).

### Measures

The collection of baseline data occurred 1 week prior to the beginning of the intervention. On-task behavior was assessed in the first two sessions of the daily school timetable during mathematics lessons. On a separate day, students were assessed for muscular fitness, executive function, and episodic memory. Research assistants were not blinded to the experimental conditions at post-test.

#### Cognitive Function

Inhibition and cognitive flexibility were assessed using executive function tests provided in the National Institute of Health Toolbox Cognition Battery (Gershon et al., 2013). The measures have acceptable test–retest reliability (ICC = 0.91 and ICC = 0.92, respectively; Bauer and Zelazo, 2013). Each of the tests was administered on an iPad using version 12.4.5 or later. Students sat individually during the test and watched the instruction video, designed by the research team, prior to commencement. The Toolbox Flanker Inhibitory Control and Attention Test Ages 12+ was administered first. The test required the participant to focus on a given stimulus while inhibiting attention to flanking stimuli (i.e., arrows) on both sides of the central target stimulus. In one condition, the middle stimulus points in the same direction as the “flankers” (congruent), and in the other condition, the middle stimulus points in the opposite direction as the flankers (incongruent). The second test in the battery was the Toolbox Dimensional Change Card Sort Test Ages 12+. This measure of cognitive flexibility displays two target pictures on the screen that vary along two dimensions, i.e., shape and color. Participants received a simultaneous verbal and visual cue which required them to match a series of bivalent test pictures (e.g., yellow balls and blue trucks) to the target pictures, first according to one dimension (e.g., color) and then, after a number of trials, according to the other dimension (e.g., shape). A 2-vector scoring method is employed for the first two tests that uses accuracy and reaction time, where each of these “vectors” contribute equally to the final calculation of the uncorrected standard score.

A third test of episodic memory adapted for use with early adolescents was added to the battery (Bauer et al., 2013). Episodic memory processes require the use of some prefrontal areas of the brain that are also associated with working memory (Van der Linden et al., 2000). The Toolbox Picture Sequence Memory Test Ages 8+ involves sequences of pictured objects and activities of the theme “playing in the park.” The pictures are presented in a specific order which the participant must attempt to reproduce. A sequence of four pictures was used as a practice and on successful completion participants could begin the test. The test involved three trials with six pictures, 15 pictures, and 18 pictures. The number of adjacent pairs placed correctly provides a representation of the participant’s estimated ability in this episodic memory task. For all three tasks, the uncorrected standard scores, with normative mean equal to 100 and 15 as SD, provided an accurate gauge of improvement or decline from Time 1 to Time 2, representing an absolute change in the level of performance since the previous assessment (National Institutes of Health, 2021).

#### On-task Behavior

On-task behavior observations were conducted using the momentary time sampling procedure adapted from the Behavior Observation of Students in Schools and the Applied Behavior Analysis for Teachers (Alberto and Troutman, 2006). On-task behavior includes times when a child is actively engaged in an academic activity (e.g., reading, writing, discussing assigned work, or performing the designated task) or passively engaged (e.g., sitting quietly listening to the teacher but is not actively participating in a set task). Off-task behavior is related to behavior not associated with the task such as off-task motor (e.g., walking around the classroom), off-task verbal (e.g., talking), and off-task passive (e.g., staring out the window). Two research assistants (blinded at baseline) entered the classroom with the class, using the first 10 min to determine student attendance for the six individuals who had been selected at random using random statistical number tables. In the case where a student was absent at baseline, they were replaced with the next student on the list. The same individuals were measured at baseline and post-test. After 10 min, which accounts for time to complete the intervention at post-test, students were observed at 15-s intervals on a rotational basis over a 30-min period. Synchronized stopwatches indicated to the research assistants the time points at which to code participants as on task active, on-task passive, off-task motor, off-task passive, or off-task verbal. Final scores were reported as a percentage of time spent on-task or off-task. Our research team has previously established the inter-rater reliability for the same on-task behavior assessment (ICC = 0.84; Mavilidi et al., 2021).

#### Muscular Fitness

Muscular fitness testing began with an introductory video, designed by the research team, which was shown to each group upon entering the testing room. During fitness testing, a rule of three was used as a protocol to warm up for fitness tests, e.g., three push-ups, three squats, or 3 s plank hold for familiarity. Three trained research assistants were each assigned to a fitness test to ensure consistency of testing protocols and students progressed between tests in the order outlined. Muscular fitness outcomes were assessed for three regions of the body—*upper body* was assessed using the 90° push-up test (Meredith and Welk, 2010). Participants started in the push-up position (males on toes and females on knees) with their hands and arms at shoulder distance apart. Keeping their back straight, participants then lowered themselves to the ground until there was 90° angle at the elbows, with upper arms parallel to the floor (Meredith and Welk, 2010). The push-ups were completed in time to a metronome set at 40 beats per minute with one complete push-up every 3 s. The participant continued until they could do no more in rhythm (e.g., did not complete the last three efforts in rhythm). This test was found to have acceptable test–retest reliability in adolescents over a period of 7 days (ICC = 0.90; Lubans et al., 2011). *Lower body* muscular fitness was assessed using the 30 s maximal repetition squat to chair test that has a test–retest coefficient of (ICC = 0.73; Bohannon, 1995). Although commonly used in older adults, this test was selected over the standing long jump to examine muscular strength rather than explosive power which is not a focus during the resistance training exercises. The 30 s sit to stand test on a bench height of 44 cm utilizes standardized protocol to assess the adolescent population (Różańska-Kirschke et al., 2006). *Core* strength was assessed using the plank hold test that required students to start with the upper body supported off the ground by the elbows and forearms, and the legs straight with the weight taken by the toes. The test is over when the subject is unable to hold their back straight and the hip is lowered to the ground. This test has acceptable test–retest reliability (ICC = 0.63; Boyer et al., 2013).

#### Rating of Perceived Exertion

A uniquely designed smartphone application was downloaded by participants and used to collect *ratings of perceived exertion* (RPE) scores before and after each session. The app displayed Borg’s modified CR10 RPE scale, represented with scores of 0, nothing at all to 10, very hard (Williams, 2017). Students reported their perceived physical exertion after completing each intervention video. Reliability and validity of the Borg scale has been reported by Lamb (Lamb, 1996) to be feasible in pre-adolescents.

### Statistical Analysis

Outcomes were analyzed using linear mixed models in IBM SPSS Statistics, version 23.0 (SPSS Inc., IBM Company Armonk, NY, United States). This statistical approach is consistent with the intention-to-treat principle because missing data, assumed to be missing at random, are modeled using a likelihood-based analysis (Mallinckrodt et al., 2004; White et al., 2012). Mixed models were used to assess the impact of group, time, and the group-by-time interaction, using random intercepts to account for the clustered nature of the data (i.e., students nested in classes). Alpha levels were set at *p* < 0.05 and Cohen’s *d* was calculated to provide a measure of effect size. Outliers that were >3 *SD* from the mean were identified as strong indications of implausible post-test data and were excluded from the analysis.

## Results

Intervention completion rates per group were: CON 83%, SECT 92%, RTNC 83%, and RTCT 92%. Reasons for incompletion were public holidays (four sessions) and whole school events (two sessions).

### Participants

Participants’ mean age was 15.78 ± 0.44, and all demographics are presented in Table 1. There were no significant differences between groups, although gender imbalance can be noted in the SECT and RTCT groups. Most of the participants reported having an Australian cultural background (94%), and 6% of students identified as Aboriginal or Torres Strait Islander. A summary of findings per variable is portrayed in Tables 2, 3.

**Table 1:** Participant demographics.

Characteristics	Control group	SECT	RTNC	RTCT	Total
Participants	(*n* = 23)	(*n* = 21)	(*n* = 29)	(*n* = 24)	(*n* = 97)
Age (years)					
Mean (standard deviation)	15.78 (0.42)	15.65 (0.49)	15.90 (0.41)	15.81 (0.40)	15.78 (0.44)
Sex, *n* (%)					
Male	15 (65)	8 (38)	12 (41)	18 (75)	53 (54.6)
Female	8 (35)	13 (62)	17 (59)	6 (25)	44 (45.4)
Aboriginal or Torres Strait Islander, *n* (%)					
Yes, *n* (%)	3 (13)	2 (10)	0 (0)	1 (4)	6 (6)
No, *n* (%)	20 (87)	19 (90)	29 (100)	23 (96)	91 (94)

CON, control condition; SECT, sedentary with cognitive training; RTNC, resistance training without cognitive training; and RTCT, resistance and cognitive training.

**Table 2:** Summary of outcome measures.

	Control group		SECT		RTNC		RTCT	
Variable	Baseline Mean (95% CI)	Post-test Mean (95% CI)	Baseline Mean (95% CI)	Post-test Mean (95% CI)	Baseline Mean (95% CI)	Post-test Mean (95% CI)	Baseline Mean (95% CI)	Post-test mean (95% CI)
Cognitive function								
Flanker test (inhibition)	103.18 (99.73, 106.63)	104.15 (100.58, 107.72)	106.55 (102.98, 110.11)	109.44 (105.73, 113.15)	105.79 (102.74, 108.85)	108.07 (105.06, 111.07)	107.90 (104.28, 111.52)	112.00 (108.25, 115.66)
Card sort change test (cognitive flexibility)	103.91 (98.85, 108.97)	103.65 (98.46, 108.84)	104.09 (98.87, 109.31)	106.21 (100.68, 111.73)	111.36 (106.90, 115.82)	110.21 (105.80, 114.61)	111.20 (105.90, 116.50)	113.99 (108.59, 119.39)
Picture sequence memory test (episodic memory)	106.41 (100.67, 112.15)	99.41 (93.46, 105.35)	108.93 (102.95, 114.91)	105.13 (98.77, 111.49)	108.82 (103.69, 113.95)	111.69 (106.69, 116.69)	114.35 (108.33, 120.367)	113.94 (107.68, 120.20)
On-task behavior								
On-task behavior	61.67 (46.54, 76.80)	45.95 (30.23, 61.68)	63.63 (48.51, 78.77)	19.66 (1.47, 37.86)	65.15 (50.08, 80.21)	57.61 (40.42, 74.79)	50.42 (35.29, 65.55)	79.97 (64.24, 95.70)
Off-task behavior	38.33 (23.27, 53.39)	54.05 (38.40, 69.71)	36.37 (21.24, 51.50)	80.34 (62.14, 98.53)	34.83 (19.83, 49.83)	42.38 (25.27, 59.50)	49.58 (34.52, 64.64)	20.04 (4.38, 35.69)
Muscular fitness								
90° Push up test	14.10 (10.57, 17.63)	15.54 (11.97, 19.10)	17.06 (13.05, 21.07)	21.72 (17.67, 25.77)	16.23 (13.04, 19.41)	17.52 (14.36, 20.69)	15.66 (11.96, 19.36)	17.47 (13.83, 21.10)
Squat to chair test	18.91 (16.59, 21.23)	23.28 (20.96, 25.60)	21.94 (19.41, 24.47)	24.60 (22.02, 27.17)	21.91 (19.84, 23.97)	24.47 (22.43, 26.50)	19.99 (17.67, 22.30)	24.14 (21.89, 26.39)
Plank hold test	78.06 (59.32, 96.79)	83.38 (63.90, 102.85)	85.18 (64.10, 106.26)	78.37 (56.99, 99.76)	81.56 (64.68, 98.44)	64.98 (48.26, 81.69)	85.41 (66.45, 104.37)	87.48 (68.75, 106.22)

CON, control condition; SECT, sedentary with cognitive training, RTNC, resistance training without cognitive training, and RTCT, resistance training with cognitive training.

**Table 3:** Adjusted difference between groups (post-test—baseline).

	CON—SECT			CON—RTNC			CON—RTCT			SECT—RTNC			SECT—RTCT			RTNC—RTCT		
Variable	Mean change (95% CI)	*p*	Cohen’s *d*	Mean change (95% CI)	*p*	Cohen’s *d*	Mean change (95% CI)	*p*	Cohen’s *d*	Mean change (95% CI)	*p*	Cohen’s *d*	Mean change (95% CI)	*p*	Cohen’s *d*	Mean change (95% CI)	*p*	Cohen’s *d*
Cognitive function																		
Flanker test (inhibition)	−1.93 (−5.86, 2.00)	0.332	0.49	−1.31 (−4.81, 2.19)	0.458	0.06	−3.09 (−6.93, 0.75)	0.113	0.43	0.62 (−3.04, 4.28)	0.737	0.38	−1.16 (−5.15, 2.83)	0.563	0.03	−1.78 (−5.34, 1.78)	0.322	0.33
Card sort change test (cognitive flexibility)	−2.27 (−7.54, 2.81)	0.365	0.00	0.90 (−3.55, 5.34)	0.688	0.20	−3.04 (−7.92, 1.83)	0.217	0.39	3.27 (−1.58, 8.11)	0.183	0.17	−0.68 (−5.92, 4.56)	0.797	0.34	−3.94 (−8.46, 0.58)	0.086	0.66
Picture sequence memory test (episodic memory)	−3.20 (−11.99, 5.58)	0.470	0.18	−9.87 (−17.71, −2.03)	**0.014**	0.72	−6.60 (−15.21, 2.02)	0.132	0.35	−6.67 (−14.92, 1.59)	0.112	0.56	−3.39 (−12.39, 5.61)	0.456	0.24	3.28 (−4.80, 11.36)	0.422	0.26
On-task behavior																		
On-task behavior	28.25 (0.48, 56.03)	**0.046**	0.84	−8.18 (−35.52, 19.16)	0.548	0.24	−45.26 (−71.50, −19.03)	**0.001**	1.23	−36.44 (−65.26, −7.61)	**0.015**	1.48	−73.51 (−101.29, −45.74)	**<0.001**	2.52	−37.09 (−64.42, −9.75)	**0.009**	1.34
Off-task behavior	−30.18 (−57.92, 2.44)	**0.034**	0.84	8.17 (−19.13, 35.47)	0.548	0.24	45.26 (19.05, 71.47)	**0.001**	1.23	38.35 (9.57, 67.13)	**0.010**	1.48	75.44 (47.71, 103.19)	**<0.001**	2.53	37.09 (9.79, 64.40)	**0.009**	1.34
Muscular fitness																		
90° Push up test	−3.23 (−6.65, 0.20)	0.064	0.51	0.14 (−3.00, 3.30)	0.931	0.26	−0.37 (−3.73, 2.99)	0.826	0.06	3.37 (0.20, 6.54)	**0.038**	0.73	32.85 (−0.53, 6.24)	0.097	0.39	−0.51 (−3.61, 2.59)	0.744	0.26
Squat to chair test	1.71 (−1.36, 4.79)	0.269	0.46	1.80 (−1.04, 4.65)	0.210	0.33	0.22 (−2.75, 3.18)	0.884	0.06	0.09 (−2.80, 2.98)	0.950	0.40	−1.50 (−4.50, 1.51)	0.324	0.37	−1.59 (−4.36, 1.19)	0.258	0.34
Plank hold test	12.12 (−10.44, 34.68)	0.288	0.59	21.90 (1.31, 42.50)	**0.037**	0.90	3.24 (−18.30, 24.78)	0.765	0.11	0.78 (−11.37, 30.93)	0.359	0.57	−8.88 (−30.96, 13.20)	0.359	0.26	−18.66 (−38.72, 1.39)	0.068	0.62

CON, control condition; SECT, sedentary with cognitive training; RTNC, resistance training without cognitive training; and RTCT, resistance training with cognitive training. Significant values have been marked as bold. Four outliers (three outliers in tests of cognitive flexibility, one outliers in tests of inhibition) were outside three standard deviations of the mean and were removed from the data set.

### Cognitive Function

No significant group-by-time effects were observed for inhibition or cognitive flexibility. A significant group-by-time effect was observed for participants’ episodic memory between the CON and RTNC groups (−9.87 units, 95% CI: −17.71 to −2.03, *p* = 0.014, *d* = 0.72).

### On-task Behavior

Significant group-by-time effects were observed for participants’ on-task behavior between the CON and SECT groups (mean change = 28, 95% CI: 0.48 to 56.03, *p* = 0.046, *d* = 0.84), between CON and RTCT (mean change = −45.26, 95% CI: −71.50 to −19.03, *p* = 0.001, *d* = 1.23), between SECT and RTNC (mean change = −36.44, 95% CI: −65.26 to −7.61, *p* = 0.015, *d* = 1.48), between SECT and RTCT (mean change = −73.51, 95% CI: −101.29 to −45.74, *p* = 0.001, *d* = 2.52), and RTNC and RTCT (mean change = −37.09, 95% CI: −64.42 to −9.75, *p* = 0.009, *d* = 1.34).

### Muscular Fitness

Significant group-by-time effects were shown for participants’ upper body muscular fitness as measured using the 90° push-up test, with the SECT group outperforming the RTNC group (mean change = 3.37 repetitions, 95% CI: 0.20–6.54, *p* = 0.038, *d* = 0.73). In addition, the CON group outperformed the RTNC group in core muscular fitness as measured using the plank hold test (mean change = 21.90 s, 95% CI: 1.31–42.50, *p* = 0.037, *d* = 0.90). There were no significant findings for lower body muscular endurance.

### Rate of Perceived Exertion

RPE for the sedentary groups of CON (*M* = 1.3) and SECT (*M* = 1.7) reflect scores on the scale that indicate very light to light intensity of physical activity (Table 4). RPE scores for RTNC (*M* = 2.25) and RTCT (*M* = 2.16) indicate an exertion rate between light and moderate physical activity.

**Table 4:** Rate of perceived exertion scores measured using Borg’s modified CR10 RPE scale.

Characteristics	Control group	SECT	RTNC	RTCT	Total
RPE scores					
Mean (standard deviation)	1.3 (2.58)	1.7 (1.25)	2.25 (2.11)	2.16 (2.00)	1.9 (1.89)

CON, control condition; SECT, sedentary with cognitive training; RTNC, resistance training without cognitive training; and RTCT, resistance training with cognitive training. Scores range from 0 = nothing at all, to 10 = very hard.

## Discussion

This study was designed to investigate the impact of classroom activity breaks involving body weight resistance exercise with and without additional cognitive demand on adolescents’ cognitive function, on-task behavior, and muscular fitness. We hypothesized that participants assigned to the RTCT group would experience larger improvements in cognitive functions than participants in the RTNC, SECT, and CON groups. We also predicted that participants in the RTNC and SECT groups would experience larger improvements in cognitive function than participants in the CON group. We hypothesized that participants in the RTCT and the RTNC group would spend more time on task compared to participants in the SECT and CON groups. Finally, we hypothesized that participants in both resistance training groups would experience improvements in muscular fitness. The resistance training group without cognitive demand significantly improved their episodic memory in comparison with the control group. However, no group-by-time effects were observed for inhibition or cognitive flexibility. Both resistance training groups improved their on-task behavior in comparison with the control group.

Classroom-based resistance training with and without cognitive training did not lead to improvements in adolescents’ inhibition and cognitive flexibility. Our findings align with a meta-analysis that reported chronic physical exercise to have a non-significant effect (*d* = 0.14) on executive functions (Verburgh et al., 2014). Our findings align with a meta-analysis that reported chronic physical exercise to have a non-significant effect (*d* = 0.14) on executive functions (Verburgh et al., 2014). More recently, a meta-analysis, focused on adolescents and young adults found conflicting results. In the analysis by Haverkamp et al. (2020), chronic interventions were shown to improve cognitive flexibility (ES = 0.19) and attention (ES = 0.50), with improvements to working memory also evident (Haverkamp et al., 2020). Chronic exercise programs typically include multiple training sessions per week for a longer period (typically spanning between 6 and 30 weeks). Largely, most chronic studies are conducted outside of the classroom or if inside the classroom, the intervention is run as an active classroom, with students simultaneously moving and learning, rather than during an active break (Kamijo et al., 2011; van der Niet et al., 2016). Interestingly, another study reporting a significant positive effect of chronic exercise on executive functions was a study of overweight children. Compared with adolescents of normal weight, obese individuals present lower cognitive indices (Smith et al., 2011) particularly in tests of executive function (Verburgh et al., 2014). Interventions designed to improve executive functions will find the greatest improvements in participants with initially poorer executive functions (Diamond and Lee, 2011) emphasizing the need for future research to focus investigations on a healthy adolescent population.

Effects were observed for episodic memory, as evidenced by significant improvements in the resistance training with no cognitive training group compared to the control group. Episodic memory relies on a set of mental processes involving encoding, storage, and retrieval of internal or external information (Squire, 2004). Research has demonstrated that prefrontal areas of the brain involved in working memory are also involved in a wide range of tasks including episodic memory (Van der Linden et al., 2000). To the best of our knowledge, this is the first study of its kind to examine the effects of resistance training on episodic memory in adolescents. Chronic training studies involved in a systematic review did not provide compelling evidence for a positive effect of resistance exercise on memory function and of the studies included, only one worked with a population younger than 50 years of age (Loprinzi et al., 2018). One example of the effectiveness of body weight resistance training was evidenced in a 52-week program delivered to a healthy older adult population (Best et al., 2015). Improvements to episodic memory function at the 1-year post intervention period were achieved but only by the group that engaged in twice-weekly resistance training sessions as opposed to once-weekly resistance training. It may be, that for the adolescent population a shorter (4 week) and more condensed (three times per week) exposure to resistance training promotes short-term improvements to episodic memory and longer interventions would be required to explore sustained improvements.

Although we did not find evidence for the chronic effect of resistance training on executive functions, improvements in on-task behavior reflect a potential acute effect for inhibition. Our measure of on-task behavior reflects an individual’s ability to ignore distractions and maintain focus on the intended task, which is a key characteristic of inhibition (Diamond, 2013). The effects for on-task behavior aligned with our hypotheses. While both resistance training groups improved, the resistance training with cognitive training group significantly outperformed all other groups. On-task behavior has been shown to contribute to academic outcomes and is displayed as behaviors that may promote learning in the classroom, e.g., concentrating on tasks assigned by the teacher (Riley et al., 2016). The positive effect of classroom activity breaks on children’s on-task behavior has been well established. For example, results of a meta-analyses showed classroom-based physical activity have a positive effect on improving students’ on-task behavior in the classroom (*d* = 0.60; Watson et al., 2017). Previous research from those involved in this study report similar improvements in on-task behavior evoked by physical activity breaks (Mavilidi et al., 2020, 2021). In particular, the Burn 2 Learn study (Leahy et al., 2019), conducted with senior school students aged 16–18 reported students being more actively engaged during classroom-based lessons after participation in HIIT sessions (*p* = 0.042, *d* = 0.43; Mavilidi et al., 2021). Students in the Burn 2 Learn program participated in two high-intensity interval training (HIIT) sessions per week that were delivered using a specifically designed phone application (Kennedy et al., 2020; Mavilidi et al., 2021). Similar to the current study, the Burn 2 Learn sessions included a high percentage of resistance training exercises (combined with aerobic activity). It is important to note the majority of Burn 2 Learn sessions were not explicitly designed to include additional demand (although the intervention did include an option for cognitively demanding high intensity exercise known as Brain HIIT). Consistent with our findings, the Burn 2 Learn program did not have a chronic effect on participants’ executive functions (i.e., working memory and inhibition) at the primary end-point of the study (6 months; Lubans et al., 2021).

Contrary to our hypotheses, the sedentary groups outperformed the resistance training groups in terms of changes in muscular fitness. As noted by Stricker et al. in their recent Clinical Report on Resistance Training for Children and Adolescents, strength gains can be achieved with different types of resistance training (including body weight exercises) for a minimum duration of 8 weeks with a frequency of 2–3 times per week. While studies involving shorter durations (e.g., 6 weeks) have reported improvements in adolescents’ muscular fitness, these studies have involved higher levels of training intensity (Gorostiaga et al., 1999) or an increased frequency of sessions (Myer et al., 2005). As such, the training load prescribed in the current study was insufficient to induce gains in muscular fitness in the study population.

### Strengths and Limitations

Overall, this study offers a unique contribution to the field by combining resistance training and cognitive training using a Latin square study design. The four groups were required to separate the effects often seen when examining physical activity delivered with and without cognitive demand. To our knowledge, this is the first study to examine the effects of cognitively engaging physical activity with adolescents.

Despite these study strengths, there are some limitations that should be noted. First, we were unable to conduct regular face-to-face fidelity checks due to COVID-19 restrictions. The infrequent fidelity checks might also have impacted the RPE scores that showed the resistance training exercises did not meet the target of moderate-to-vigorous intensity. Second, participants were randomized by class to the four study groups. Future studies are encouraged to include a larger number of classes (i.e., clusters) or randomize students at the individual levels. Third, we did not include a measure of working memory in our battery of cognitive tests. We were unable to include the working memory measure from the NIH Toolbox as it requires the use of Bluetooth keyboards connected with devices, which was problematic in a small classroom setting. Fourth, without a measure of height and weight to determine body composition we were unable to determine if weight status moderated the effect of the intervention. Finally, we assessed participants’ muscular fitness using three field-based tests (i.e., push-up, sit-to-stand, and plank tests). These tests were selected based on their existing reliability and validity and their alignment with our body weight resistance training protocols. It is important to note that these measures assess muscular endurance not strength, and test performance is influenced by participants’ motivation. For example, it was noted by the assessment team that a number of students dropped out of the plank hold test due to boredom rather than fatigue. The push-up test had its own limitations as students regularly could not complete a single push-up making small improvements difficult to calculate.

## Conclusion

In conclusion, our findings suggest that resistance training without cognitive training can improve adolescents’ episodic memory but not inhibition and cognitive flexibility. Alternatively, activity breaks both with and without cognitive demand improved adolescents’ on-task behavior. This finding is particularly important as on-task behavior is a key predictor of academic success (Mahar et al., 2006). Using resistance training as a classroom-based exercise break is novel and has been underutilized. We are wary of making definitive statements based on our initial research findings, but we believe evidence from the current study promotes further use of resistance training breaks in the classroom if particular attention is paid to the intensity of the exercise.

## Data Availability Statement

The raw data supporting the conclusions of this article will be made available by the authors, without undue reservation.

## Ethics Statement

The studies involving human participants were reviewed and approved by the University of Newcastle Human Research Ethics Committee. Written informed consent to participate in this study was provided by the participants’ legal guardian/next of kin. Written informed consent was obtained from the individual(s) for the publication of any identifiable images or data included in this article.

## Author Contributions

DL, MM, SV, DB, CH, and NR secured funding for the project. KR, DL, MM, and NR designed the intervention, designed and contributed to the administration of the cognitive and muscular fitness assessments, and were responsible for drafting the manuscript. KR and NR designed the RPE measurement protocol and phone application and were responsible for project management, school recruitment, and data collection. DL guided the statistical analysis. All authors contributed intellectually to the study design and research methodology. All authors contributed to the article and approved the submitted version.

## Funding

Funding was generously provided by the HMRI philanthropic donations, grant number: G19011491.

## Conflict of Interest

The authors declare that the research was conducted in the absence of any commercial or financial relationships that could be construed as a potential conflict of interest.

## Publisher’s Note

All claims expressed in this article are solely those of the authors and do not necessarily represent those of their affiliated organizations, or those of the publisher, the editors and the reviewers. Any product that may be evaluated in this article, or claim that may be made by its manufacturer, is not guaranteed or endorsed by the publisher.
